# The nephroprotective potential of russelioside B isolated from *Caralluma quadrangula* in gentamicin-induced acute kidney injury via modulation of SIRT-1 pathway

**DOI:** 10.1038/s41598-025-29874-7

**Published:** 2025-12-13

**Authors:** Mawada Mohamed Ali, Asmaa K. Al-Mokaddem, Essam Abdel-Sattar, Riham A. El-Shiekh, Samira H. Aljuaydi, Iman B. Shaheed

**Affiliations:** 1 Department of Pathology, Faculty of Veterinary Medicine, Cairo University, Giza, 12211 Egypt; 2 Department of Pharmacognosy, Faculty of Pharmacy, Cairo University, Cairo, 11562 Egypt; 3 Department of Biochemistry and Molecular Biology, Faculty of Veterinary Medicine, Cairo University, Giza, 12211 Egypt

**Keywords:** Nephrotoxicity, Histopathology, C*ralluma quadrangular*, Russelioside B, Gentamicin, Pharmaceutics, Pharmacology, Biochemistry, Diseases, Medical research, Nephrology

## Abstract

Using nephrotoxic antibiotics such as gentamicin may result in acute renal damage. This study aimed to investigate the nephroprotective effect of russelioside B (RB) isolated from C*ralluma quadrangula* (Forssk.) N.E.Br against gentamicin-induced acute kidney injury. Twenty male rats were randomly distributed into four groups (five animals each). Normal, Gentamicin, Genta + 50RB, and Genta + 75RB groups. The RB was taken orally for 3 days at a dose of 50 and 75 mg/Kg B.wt) before injection with gentamicin, followed by the same dose along with gentamicin injection (I/P) for 7 consecutive days. At the end of the study, kidneys and blood samples were collected. The blood samples were tested for BUN and Creatinine. Tissue samples were tested for SOD, MDA, and NO. Genes expression of IGF-1, iNOS and Bcl2 by RT-PCR were tested. Histopathological evaluation was performed along with immunohistochemistry for SIRT1, NQO1, Nrf-2, TNF-α, and NF-қB. RB treated groups showed reduction in serum BUN and creatinine levels. Along with reduced MDA and nitric oxide while there was an elevation in SOD, SIRT1, NQO-1, Nrf2, and IGF-1 with a decrease in TNF-α, NF-қB and iNOS. Confirming the anti-oxidant and anti-inflammatory properties of RB. RB treatment resulted in elevation in the anti-apoptotic protein Bcl2 and reduction in BAX confirming the anti-apoptotic potential of RB. Gentamicin-induced histopathological alterations were also alleviated with RB treatment. The findings of the study proved the nephroprotective potential of RB.

**Supplementary Information:**

The online version contains supplementary material available at 10.1038/s41598-025-29874-7.

## Introduction

Acute kidney injury (AKI) is a condition that leads to a decrease in renal output, requires intensive medical care for a long period, and could even cause death so it’s considered a serious illness^1^. AKI could also affect other organs as it could cause rising in the hepatic enzymes, infiltration with leukocytes, congestion, and hepatic cellular necrosis which is called remote organ injury^2^. Acute injury of the kidney happens within a few days or hours. It could occur due to different reasons and antibiotic usage is one of them^3^.

An example of an antibiotic that causes nephrotoxicity is aminoglycosides, such as gentamicin. This group is absorbed by the proximal tubules and remains in them for a long period, which causes nephrotoxicity^4^.

Although gentamicin is a powerful treatment for gram-negative bacteria, it has been found that about 30% of patients who receive it for more than seven days experience signs of nephrotoxicity^2,5^.

Numerous studies have demonstrated that administering high doses of gentamicin can result in cellular injury, and necrosis due to the production of reactive oxygen species (ROS) caused by the drug. These ROS can lead to peroxidation of membrane lipids, denaturation of proteins, and damage to DNA^6^.

These ROS produced by gentamicin lead to histopathological alterations, increased oxidative parameters, urea and creatinine. The drug-induced toxicity can indeed be mediated by oxidative stress, resulting in a significant cellular redox imbalance and subsequent oxidative damage. One of the critical indicators in evaluating oxidative stress is malondialdehyde (MDA), which offers a reflection of the body’s overall oxidative potential. Conversely, the body’s defense mechanisms include antioxidant enzymes such as superoxide dismutase (SOD), which play a vital role in protecting tissues from the harmful effects of reactive oxygen species (ROS) and preventing oxidative injury^7^.

Russelioside B (RB) is a pregnane glycoside isolated from several *Caralluma* species^8–10^. RB is an attractive natural compound in the treatment of several conditions indicating its efficacy as anti-ulcer, anti-obesity, anti-diabetic, and anti-arthritis agent^11–13^. In a previous study, Caralluma extract significantly reduced the renal damage caused by cisplatin and gentamicin^14^.

In the current study we aimed to investigate the role of russelioside B as a nephroprotective agent highlighting its anti-inflammatory, antioxidant and anti-apoptotic mechanisms.

## Result

### Russelioside B improved kidneys’ gross pathology, KW/BW ratio and restored the normal kidneys’ function indices

**Fig. 1:**
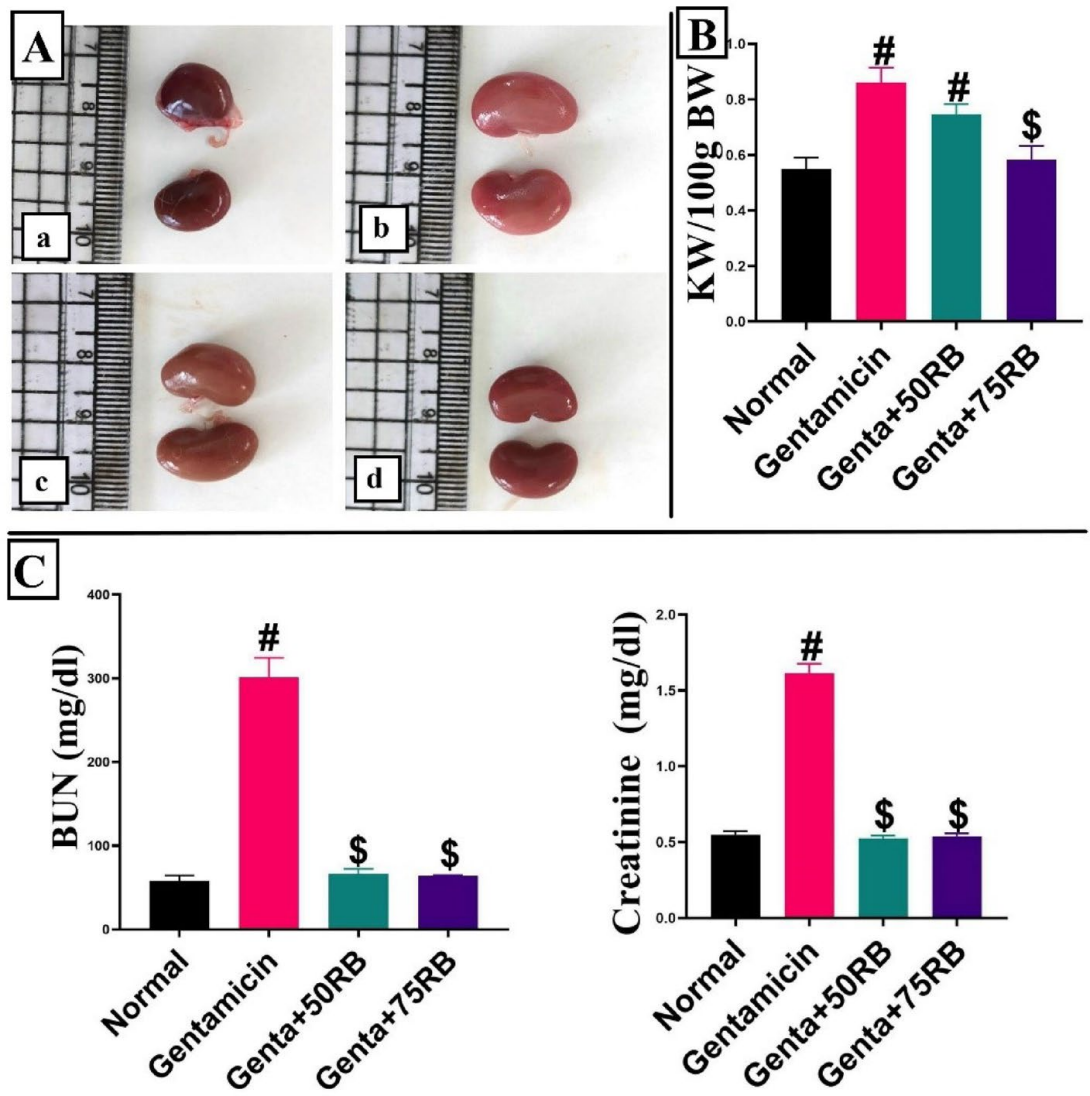
(**A**) Gross photos of kidneys showing the change in color in different groups (**a**) normal group, (**b**) Gentamicin group, (**c**) Genta + 50RB group, (**d)** Geta + 75RB, (**B**) chart represents KW/100 g BW. (**C**) Charts represent kidney’s function markers. Data are presented as mean ± SE Significant difference is considered at *P* < 0.05. (#) Significant from normal, ($) Significant from Gentamicin, (@) Significant from Genta + 50RB group.

The kidneys treated with gentamicin appeared pale in color, while those treated with RB showed a dose-dependent restoration to the normal reddish-brown color (Fig. 1A). The ratio of kidney weight to body weight (KW/BW) was highest in the gentamicin group, while the RB-treated groups had a lower ratio with no significant difference between the two tested doses. Additionally, there was no significant difference between the normal group and the high-dose RB group (Fig. 1B).

The group treated with gentamicin showed a significant increase in the levels of estimated serum creatinine and BUN when compared to the normal group. However, both treated groups showed an absence of significant difference when compared to the normal group (Fig. 1C).

### RB treatment enhanced renal expression of SIRT1

**Fig. 2:**
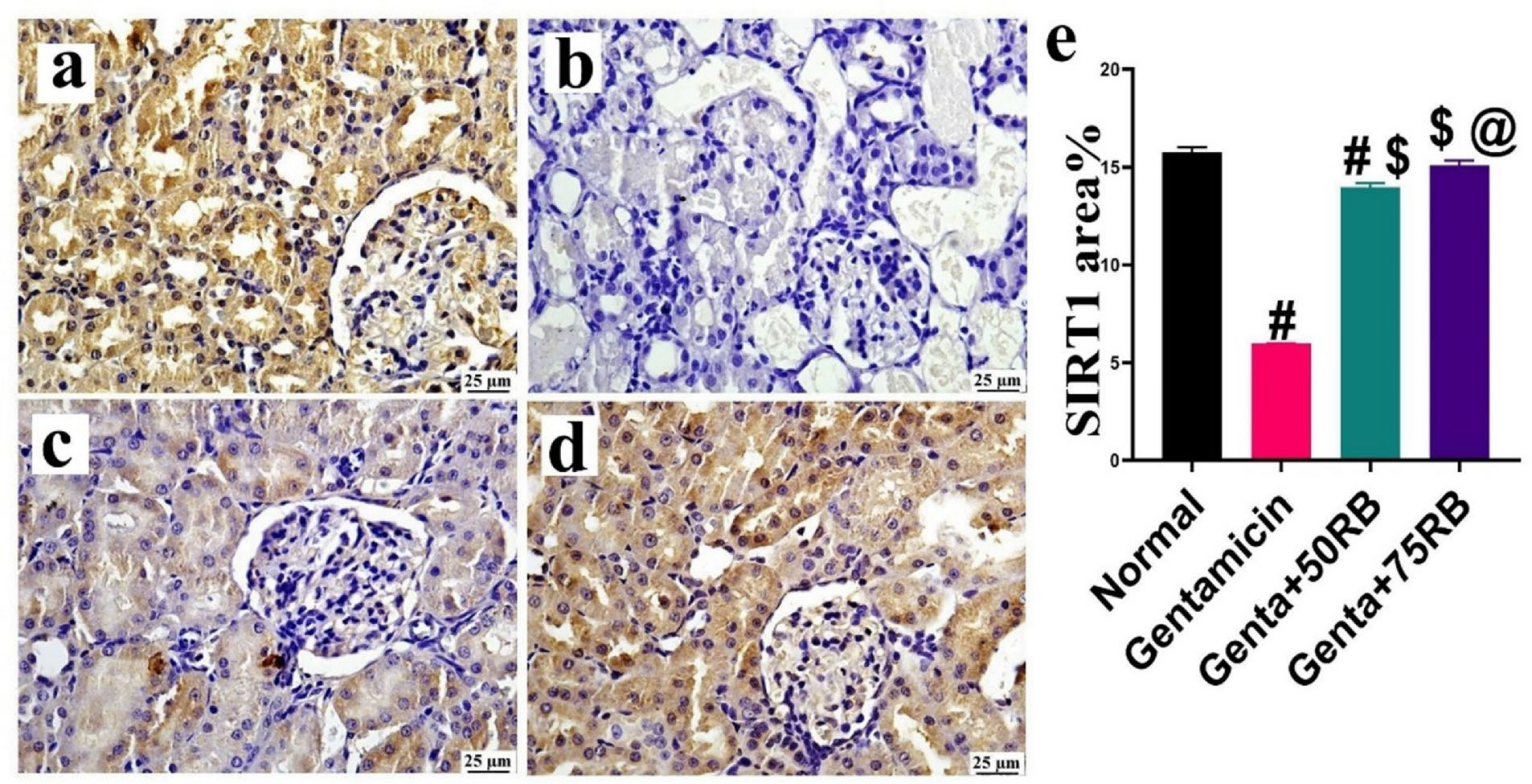
Photomicrographs of kidneys (Immune staining) showing SIRT1 expression (**a**) normal group: high expression, (**b**) Gentamicin group: mild expression, (**c**) Genta + 50RB and (**d**) Genta + 75RB groups showing moderate SIRT1 expression and (**e**) Chart represents SIRT1 quantification as area percentage. Data are presented as mean ± SE. Significant difference is considered at *P* < 0.05. (#) Significant from normal, ($) Significant from Gentamicin, (@) Significant from Gentamicin + 50RB group.

As illustrated in Fig. 2. The normal group recorded the highest SIRT1 expression, while the Gentamicin had the lowest. The treatment with RB showed a significant increase in the level of SIRT1 expression. However, there was no significant difference between the groups treated with different doses of RB. The group treated with the high dose of RB did not show any significant difference compared to the normal group.

### RB improved the oxidative status of gentamicin intoxicated kidneys

**Fig. 3:**
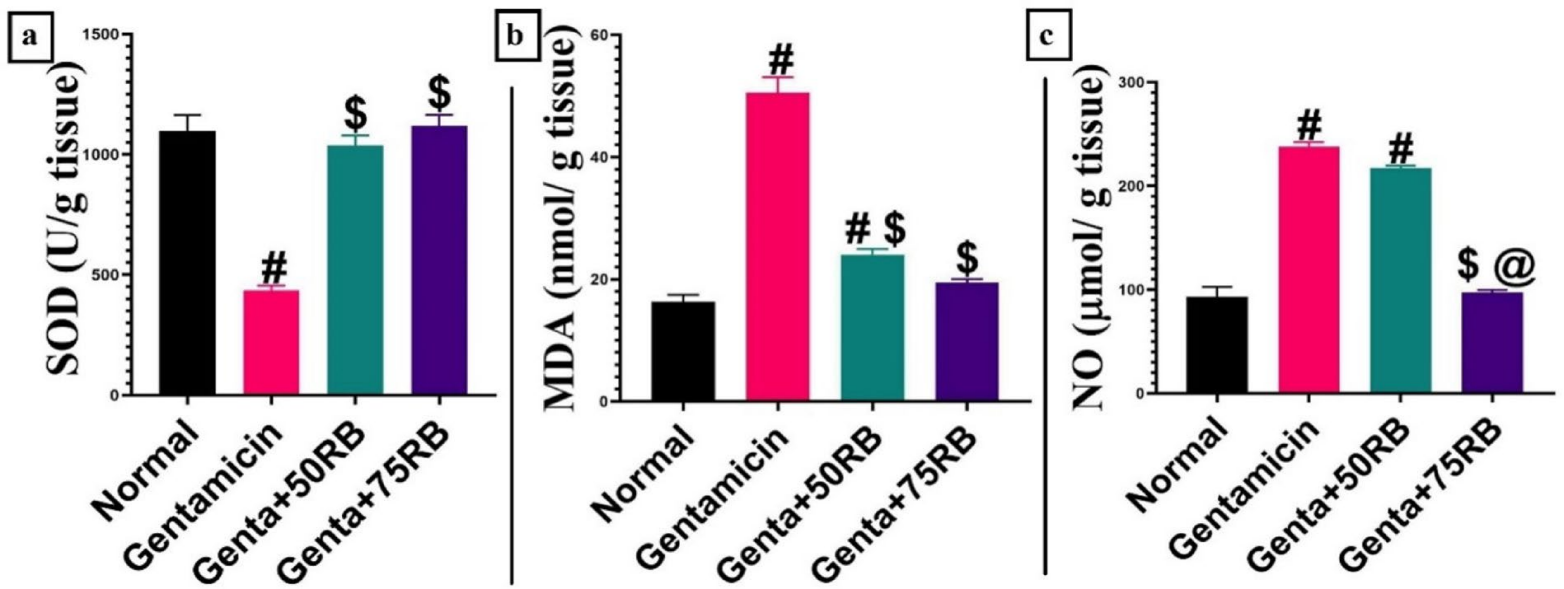
Charts represent oxidative stress markers (**a**) Superoxide dismutase (SOD), (**b**) Malonaldehyde (MDA), and (**c**)Nitic oxide (NO). Data are presented as mean ± SE. significant difference is considered at *P* < 0.05. Significant difference is considered at *P* < 0.05. (#) Significant from normal, ($) Significant from Gentamicin, (@) Significant from Gentamicin + 50RB group.

There was a notable decrease in the estimated SOD in the Gentamicin group, while there was an increase in SOD expression in both groups treated with RB, without a significant difference from the normal group. The MDA levels were the highest in the Gentamicin group, but there was a significant decrease in MDA levels in both RB-treated groups. The Genta + RB75 group did not show any significant difference from the normal group. In comparison with the normal group, the gentamicin-intoxicated group showed a significant elevation in NO, which was significantly reduced in the Genta + RB75-treated group, and only a numerical, non-significant reduction was detected in Genta + RB50 group (Fig. 3).

**Fig. 4:**
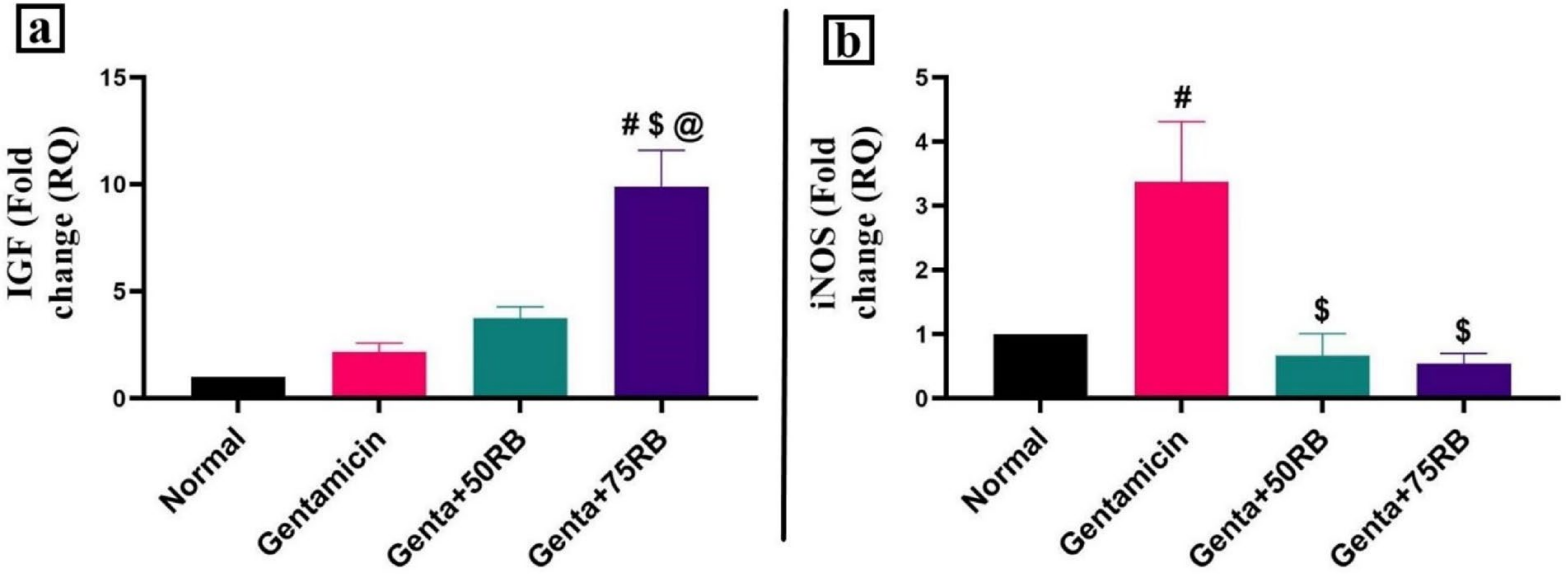
Quantitative RT-PCR evaluation of gene expression in different groups (**a**) IGF-1 gene expression, and (**b**) iNOS gene expression. Data are presented as mean ± SE. Significant difference is considered at *P* < 0.05. (#) Significant from normal, ($) Significant from Gentamicin, (@) Significant from Genta + 50RB group.

As depicted in Fig. 4a, IGF-1 showed a non-significant gradual increase in Gentamicin and Genta + 50RB groups and a significant increase in Genta + RB75 group. The gene expression of iNOS (Fig. 4b) showed a discernible uprise in the Gentamicin group opposite the control group. iNOS began to regress significantly in the group treated with low dose of RB opposite the control group with a further significant decline in 75RB treated group opposite the 50RB treated group.

**Fig. 5:**
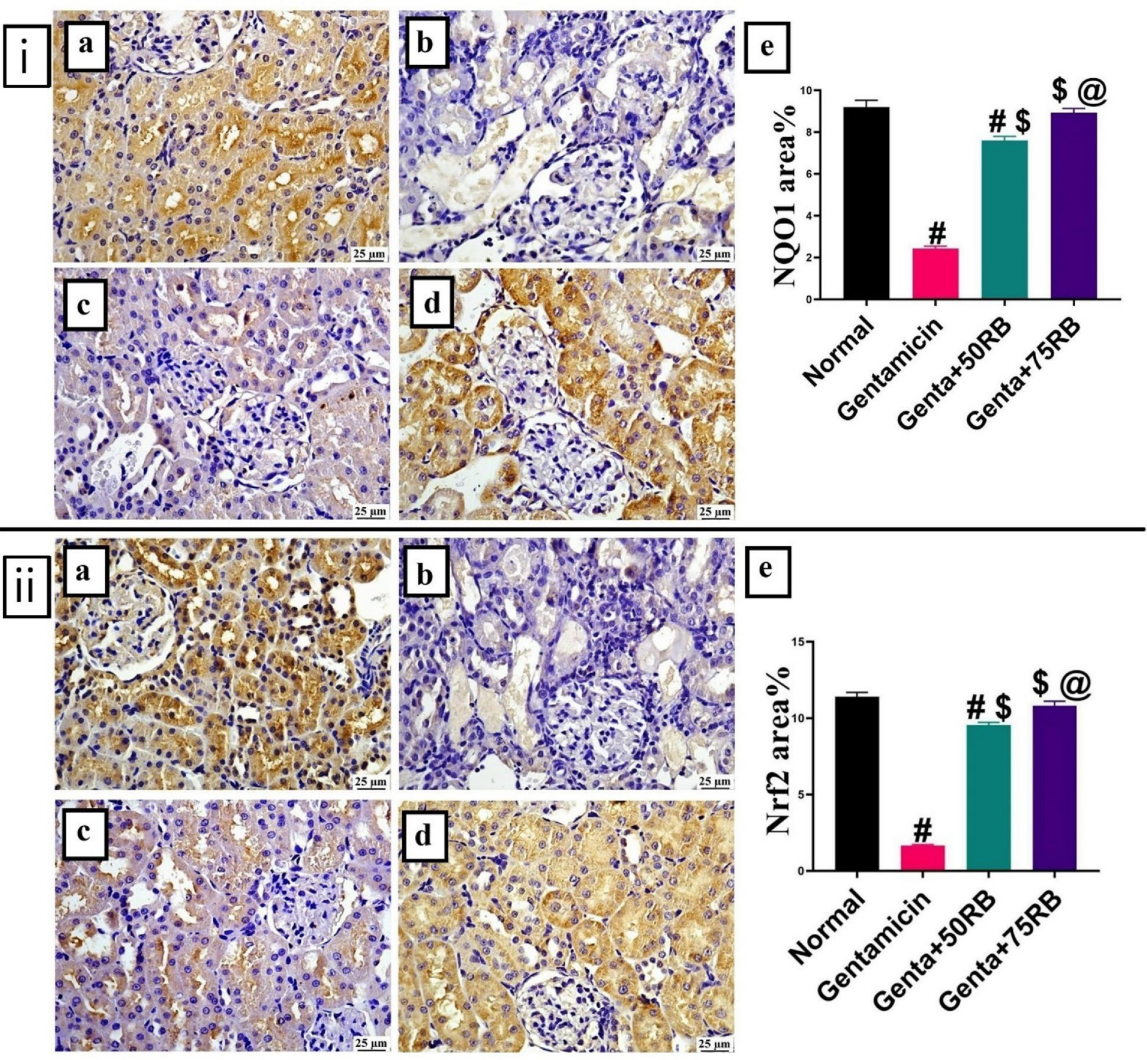
Photomicrographs of kidneys (Immune staining) showing (**i**) **NQO1 expression** and (**ii**) **Nrf2 expression** (**a**) Normal group: high expression, (**b**) Gentamicin: low expression (**c**) Genta + 50RB: moderate and (**d**) Genta + 75RB: increased expression, (**e**) Charts represent quantification of expression as area percentage. Data are presented as mean ± SE. Significant difference is considered at *P* < 0.05. (#) Significant from normal, ($) Significant from Gentamicin, (@) Significant from Genta + 50RB group.

In comparison with the normal group, renal NQO1 expression was the lowest in the Gentamicin group. However, the groups treated with RB showed increased expression levels, and there was a significant difference between the two tested doses. The normal group displayed a meaningful difference when compared to the low-dose RB-treated group, while the high-dose RB-treated group showed no significant difference (Fig. 5i).

The Gentamicin group showed a significant decrease in the expression of the Nrf-2. However, there was a significant increase in its level in the groups treated with RB, with the highest value in Genta + 75RB group. In comparison with the normal group, there was a significant difference to the low dose RB-treated group, while there was no significant difference with the RB high-dose treated group (Fig. 5ii).

### RB exerted an anti-inflammatory effect against gentamicin-induced renal damage

**Fig. 6:**
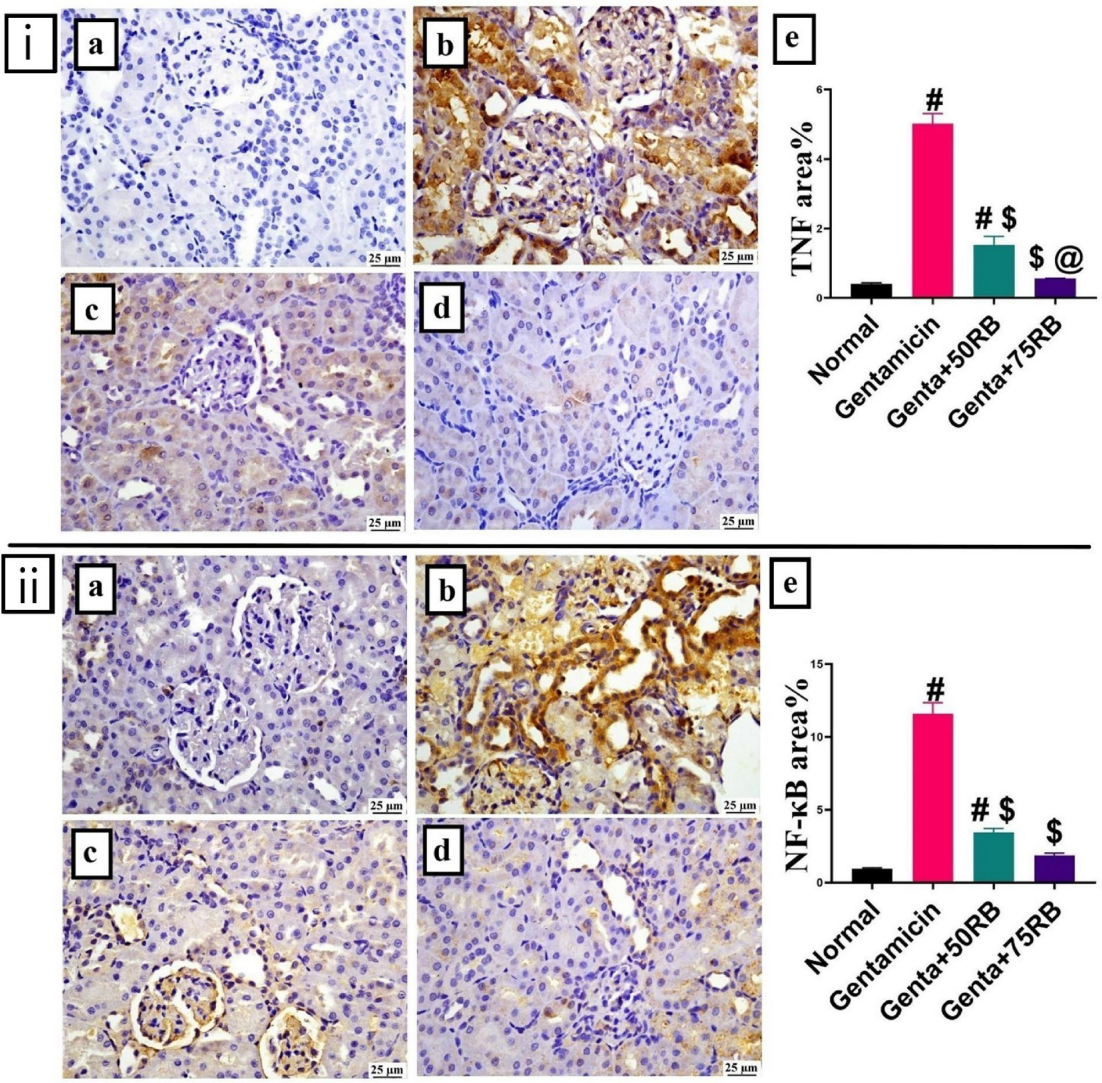
Photomicrographs of kidneys (Immune staining) showing (**i**) **TNF-α expression** and (**ii**) **NF-κB expression** (**a**) Normal group: limited expression, (**b**) Gentamicin: high expression (**c**) Genta + 50RB: moderate and (**d**) Genta + 75RB: low expression, (**e**) Charts represents quantification of expression as area percentage. Data are presented as mean ± SE. Significant difference is considered at *P* < 0.05. (#) Significant from normal, ($) Significant from Gentamicin, (@) Significant from Genta + 50RB group.

The Gentamicin group showed significant elevation in TNF-α levels when compared to the normal group. Both treated groups showed significant reduction in TNF-α expression in dose dependent manner with absence of significance between RB70-treated group and the normal group (Fig. 6i). NF-κB level was remarkably higher in the Gentamicin group compared to the other groups, while there was a significant decrease in its level in the groups treated with RB. There was no statistically significant difference between the two tested doses of RB. When comparing the two treated groups with the normal group, RB low dose treated group showed a slight significance while RB-high dose group showed no significant difference (Fig. 6ii).

### RB exerted an anti-apoptotic effect against gentamicin-induced renal damage

**Fig. 7:**
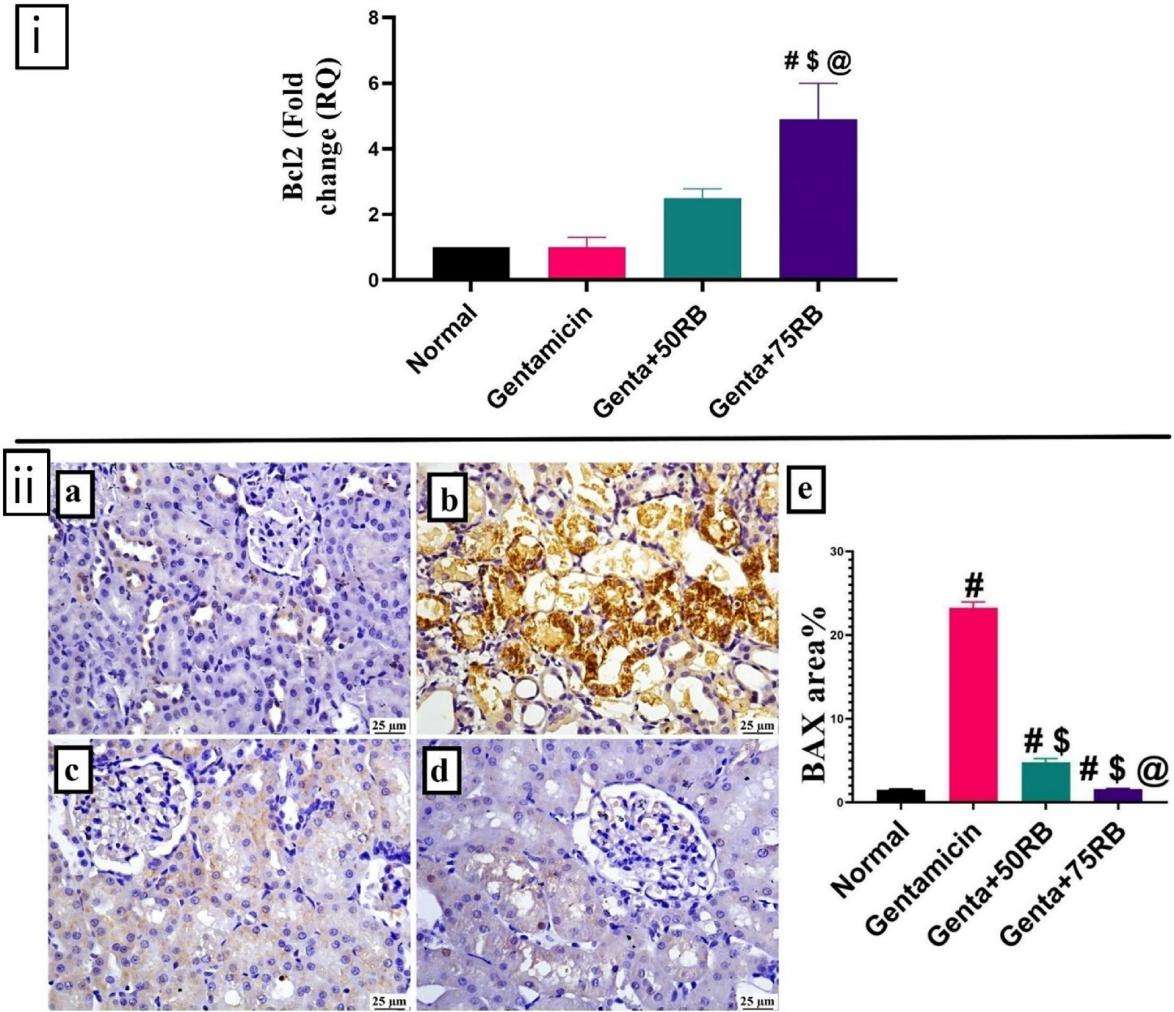
(**i**) **Quantitative RT-PCR evaluation of BCl2 gene expression**. Data are presented as mean ± SE. Significant difference is considered at P < 0.05. (#) Significant from normal, ($) Significant from Gentamicin, (@) Significant from Genta + 50RB group. **(ii) Photomicrographs of kidneys (Immune staining) showing BAX expression** (**a**) Normal group: limited expression, (**b**) Gentamicin: high expression (**c**) Genta + 50RB: moderate and (**d**) Genta + 75RB: low expression, (**e**) Charts represents quantification of expression as area percentage. Data are presented as mean ± SE. Significant difference is considered at *P* < 0.05. (#) Significant from normal, ($) Significant from Gentamicin, (@) Significant from Genta + 50RB group.

The group treated with gentamicin displayed a downregulated BCl2 gene expression versus the normal control group without a significant difference, whereas the treatment with 75 mg of RB mitigated this effect evidenced by a significantly upregulated BCl2 gene expression versus the group receiving just gentamicin (Fig. 7i).

As illustrated in Fig. 7ii, the immune expression of BAX was significantly elevated in Gentamicin group when compared to the normal group. Both tested doses of RB succeeded in decreasing renal BAX expression when compared to the Gentamicin group. Among both treatments, the lowest BAX value was detected in Genta + 75RB.

### RB treatment alleviated renal histopathological changes induced by gentamicin

No histopathological changes were detected in the examined kidney sections from the normal group. On the contrary, the Gentamicin group revealed marked histopathological alterations. Cortical renal tubules suffered from severe diffuse necrosis with nuclear pyknosis and karyolysis. The lumen of the tubules contained eosinophilic proteinaceous cast with the existence of some exfoliated cells. Some tubules demonstrated cystic dilatation. The interstitial tissue was heavily infiltrated with mononuclear inflammatory cells. Congestion of renal vasculature was also prominent. The renal tubules of the medulla showed degeneration and necrosis with the existence of an eosinophilic cast in their lumen and diffuse interstitial infiltrations with mononuclear inflammatory cells. The Genta + 50RB group showed a slight alleviation of gentamicin-induced renal damage; there was a mild improvement in both the cortex and medulla of the kidney in terms of the degree of degeneration and accumulation of eosinophilic cast at renal tubules, as well as the congestion of blood vessels. Focal interstitial was also noticed.

**Fig. 8:**
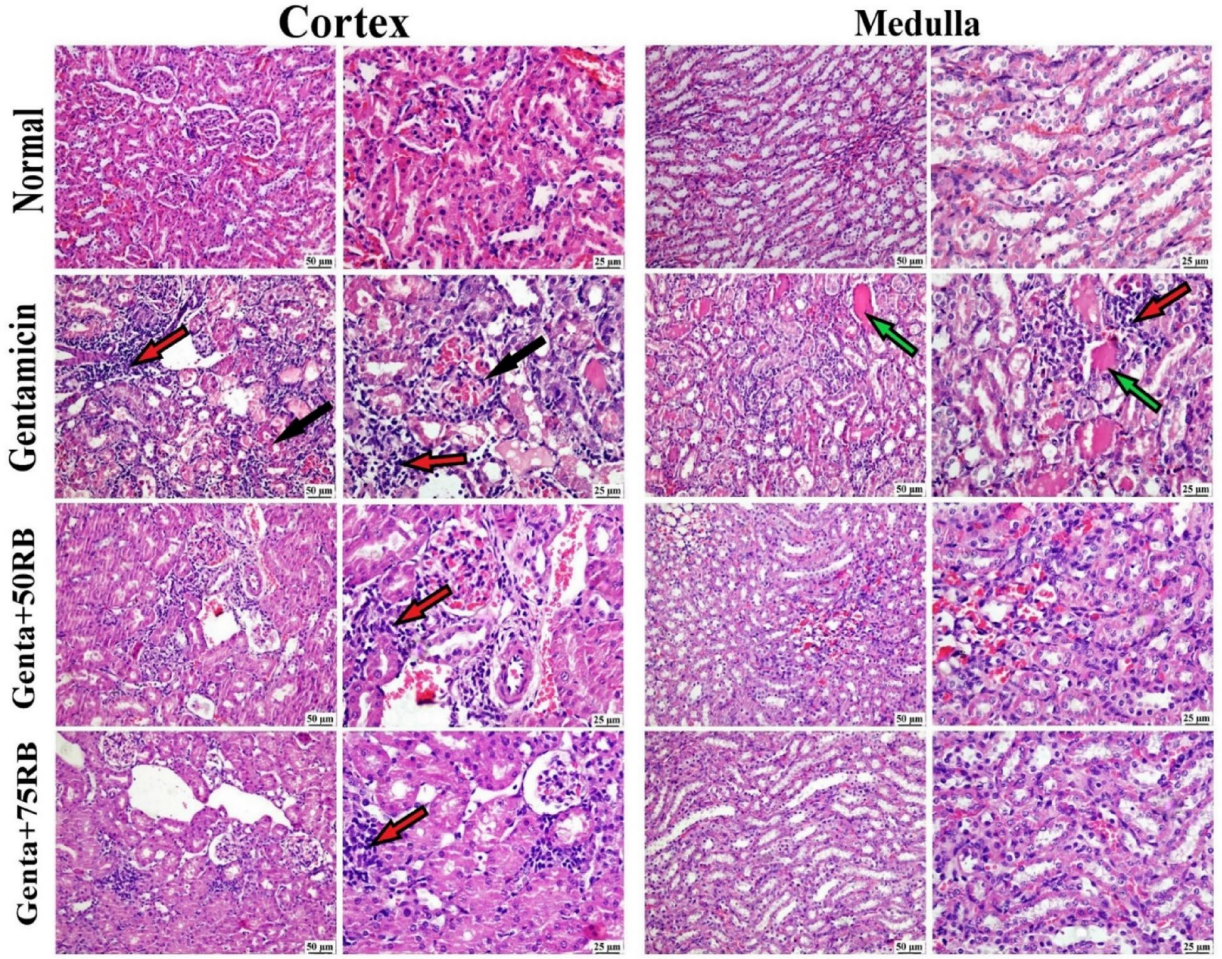
Photomicrographs of kidney (H&E) showing normal histology of both cortex and medulla in normal group, diffuse interstitial nephritis, renal tubular necrosis and cast in Gentamicin group, mild perivascular mononuclear inflammatory cells infiltration in Genta + 50RB group and mild focal aggregations of mononuclear inflammatory cells and apparently normal renal tubules in Genta + 75RB group. (Black arrows) = renal tubular necrosis, (red arrows) = inflammatory cells infiltrations, (green arrows) = renal cast.

The group treated with Genta + 75RB showed significant improvements in both the renal cortex and medulla. There were mild degenerative changes in a few tubules, but the renal cast was greatly reduced. Only minimal focal interstitial nephritis was observed in a few examined sections (Fig. 8).

**Fig. 9:**
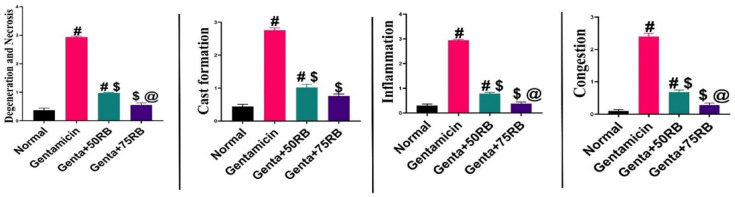
Histopathological lesion score of kidneys in the different experimental groups. Data are presented as mean ± SE. Significant difference is considered at *P* < 0.05. (#) Significant from normal, ($) Significant from Gentamicin, (@) Significant from Gentamicin + 50RB group.

All assessed histological scoring parameters (Fig. 9) indicated that the Genta + 75RB group was the best treated, as there was no significant difference between this group and the normal group for any parameter.

**Fig. 10:**
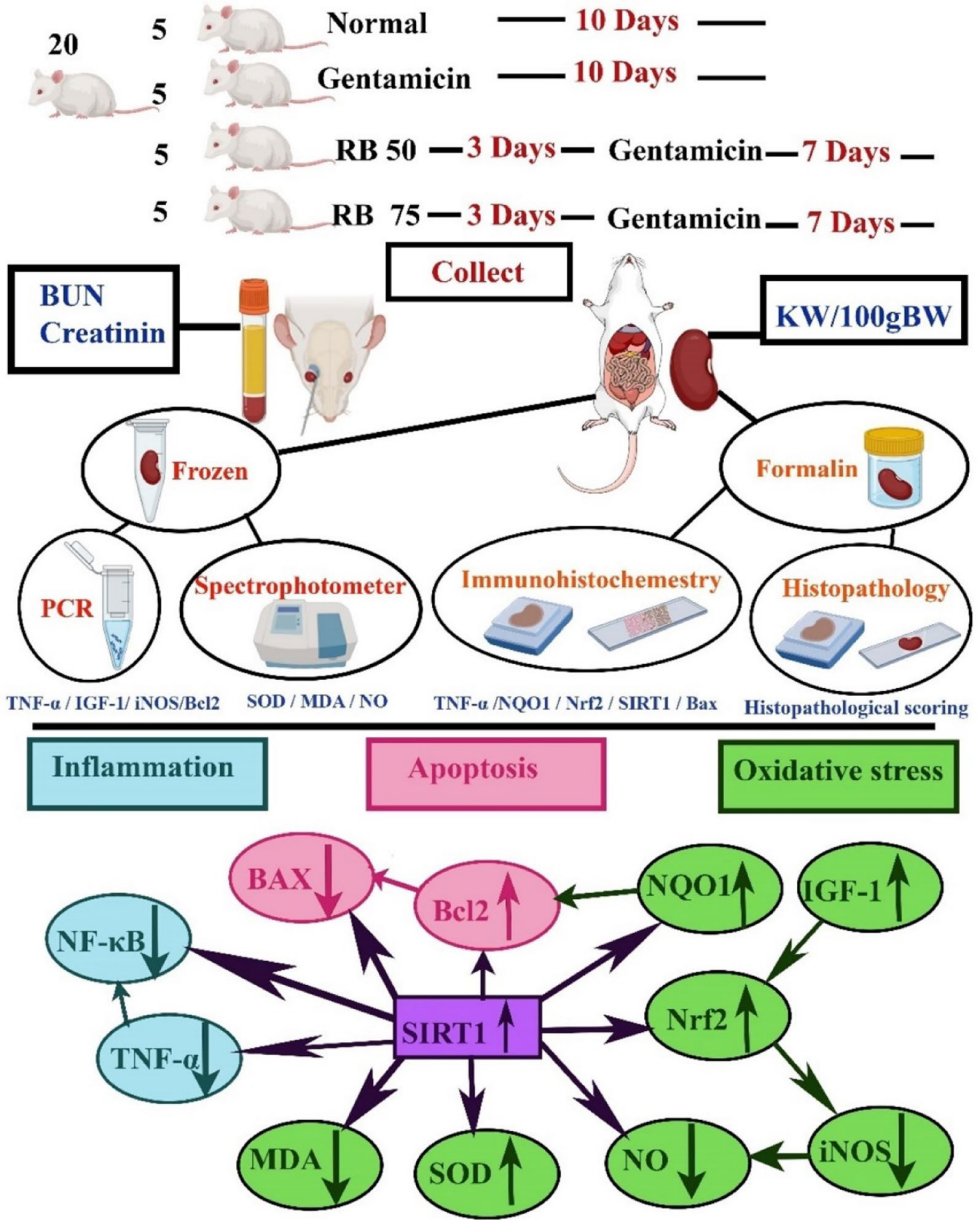
A summarization of the experimental design and the assessments used for the evaluation of the nephroprotection as well as a summarization of the effect of RB on the inflammatory status, apoptosis activity, and oxidative stress through the activation of SIRT-1 pathway.

A summarization of the effect of RB on the inflammatory status, apoptosis activity, and oxidative stress through the activation of SIRT-1 pathway (Fig. 10).

## Discussion

When animals are given gentamicin, it can cause a condition called AKI. This results in a decrease in blood flow to the kidneys, which in turn leads to a decline in the glomerular filtration rate. gentamicin mainly affects the tubular epithelium, especially the epithelium of the proximal tubules resulting in tubular cytotoxicity^15^. Research is being conducted worldwide to find a substance that can protect the kidneys and other organs with the least side effects during their functioning in the body^16^.

There is an evidence suggesting that plant extracts can prevent or reduce the severity of induced renal damage^5^. *Caralluma* species are now commonly used in traditional medicine to treat various conditions such as rheumatism, diabetes, malaria, inflammation, ulcers, kidney pain, and wounds^11,12^.

This plant is of great medical importance as it contains various compounds that have medicinal significance such as pregnane glycosides, terpenoids, flavone glycosides, and sterols. Our group has isolated Russelioside B (RB), a major pregnane glycoside, from *C. Quadrangular*^12^ to evaluate its potential nephroprotective role against gentamicin-induced renal damage.

Fauzi et al.,^3^ validated the gross examination as we recorded, in our study, the normal control negative group’s kidney was of normal reddish-brown color, meanwhile the kidneys of Gentamicin group were much paler that could be attributed to gentamicin induced nephrosis and RB treatment resulted in the restored gross appearance of kidneys.

It was shown in the^17^ study that the kidney weight to body weight ratio (KW/100 g BW) in the group treated with gentamicin was higher than in the normal group due to abnormalities of renal structure and nephrotoxicity. Our study recorded similar results, showing an increase in KW/100 g BW in the Gentamicin group, while KW/100 g BW was decreased in the groups treated with RB.

The levels of serum BUN and creatinine are used as main markers of nephrotoxicity^16^. An elevation in the levels of these markers specifies nephrotoxicity^18^. Our research revealed that the group treated with gentamicin had higher levels of BUN and creatinine in the tested serum. In contrast, the groups treated with RB showed significantly decreased levels of both parameters, ultimately reaching normal levels. This confirms the nephroprotective effect of RB.

SIRT1 is an enzyme that repairs DNA, maintains chromosomal stability, and regulates gene transcription. It also protects against acute kidney injury (AKI) by resisting oxidative stress, inflammation, and apoptosis^19,20^.

Oxidative stress can be harmful to the renal cells, leading to acute kidney injury. It occurs when there is no harmony between the production and elimination of reactive oxygen species (ROS). However, an increase in the expression of SIRT1 can reduce oxidative stress and protect renal tissue^21,22^. Our study supports these findings, as we observed a significant increase in the expression level of SIRT1 in the groups treated with RB.

It was stated by Raji-amirhasani et al.,^19^ that SIRT1 can protect against oxidative stress by increasing the production of Nrf-2, NQO1, and SOD while reducing MDA and NO. Nrf-2 and NQO1 are compounds that help in the attenuation of oxidative stress and protect tissues^18,20^. On the other hand, SOD is an important enzyme that fights against ROS and aids in the antioxidant mechanism^23^. Meanwhile, MDA and nitric oxide are products formed during the oxidative stress^24^. Our results supported these findings, as we observed an increase in the expression of Nrf-2, NQO1, and SOD, with a reduction in MDA and NO along with the increase in SIRT1 levels highlighting the role of RB in nephroprotection as a potent antioxidant.

Another oxidative marker is iNOS, the increase in the activity of iNOS leads to higher levels of nitric oxide (NO)^15^. Additionally, the activation of Nrf2 leads down regulation of iNOS^18^, which align with our findings, as the groups treated with RB showed a decrease in iNOS expression. All the previous data confirms the antioxidant potential of RB as previously described by El-Shiekh et al.,^12^.

One of the most important factors for the normal growth of renal tissue is IGF-1 as it helps in cells proliferation, differentiation, and survival^25^. Some researchers mentioned the relationship between IGF-1 and Nrf2. The deficiency in IGF-1 leads to an increase in oxidative stress as it leads to impairment in Nrf2 antioxidant pathway. So, the improvement in IGF-1 could lead to the protection of the tissue against oxidative stress by correcting the Nrf2 pathway^26^. This confirms our findings as the groups treated with RB showed an up regulation in IGF-1 expression with subsequent elevation in Nrf2 expression.

After an injury, the inflammatory response is triggered to eliminate any microbial agents and start the repairing process^19^. One of the major cytokines which is responsible for inflammatory reaction initiation is TNF-α^21^ and when it comes to AKI it’s responsible for decreasing the blood flow via vasoconstriction, infiltration of inflammatory cells, and ICAM-1 production^2^. TNF-α has a relation with NF-κB as its activation causes NF-κB up regulation ^27^. NF-κB induces some inflammatory mediators that regulate the inflammation^28^. It was declared by Raji-amirhasani et al.,^19^ that SIRT1 has an anti-inflammatory effect via inhibition of TNF-α and NF-κB cascades.

Our results were in harmony with the above-mentioned mechanism, as the groups treated with RB showed an upregulation in the expression of SIRT1 and significant decrease in the expression of both TNF-α and NF-κB. Our findings confirmed the anti-inflammatory properties of RB as previously described by Abdel-Sattar et al.,^13^; Abdel-Sattar and Ali,^29^; El-Shiekh et al., ^11,12^.

Apoptosis is the programmed cell death that’s initiated by factors such as inflammation and oxidative stress. It occurs due to a balanced process between the pro-apoptotic factors and the anti-apoptotic factors of the Bcl-2 gene family^18^. One of the factors that promotes apoptosis is BAX, a member protein of BCl-2 family that encourages the apoptosis by competing with BCl-2^30^.

It’s important to investigate the apoptosis levels in AKI as it has a fundamental role in renal lesions pathogenesis through the enhancement of BAX and the drop of Bcl-2 levels^1^.

It was mentioned by ^19^ that SIRT1 has an antiapoptotic effect through the activation of anti-apoptotic proteins of Bcl-2 and the downregulation of BAX. Meanwhile Roberts,^31^ mentioned that the more heightened BCl2 expression the more downregulated BAX activation. Additionally,^20^ declared that the over-expression of NQO1 in renal cells elevated the expression of Bcl-2.

In our results, RB-treated groups showed an elevation in the expression of both SIRT1, and NQO1 along with the increase in the Bcl-2 anti-apoptotic gene, while also expressed a significant decrease in the level of BAX. This confirms the antiapoptotic effect of RB as cited before by^11^.

Several pathological alterations were recorded in this study due to the effect of gentamicin injection. There were degenerative changes at the tubular epithelium especially in the cortex that exhibited cellular necrosis. Some tubules contained cellular and proteinaceous cast. Blood vessels and glomerular capillary tufts suffered from congestion and the interstitial tissue endured inflammatory cells infiltration. These findings go along with the results of Mohamadi et al.,^2^; Padmini and Kumar,^4^; Rizwan et al.,^32^ that confirmed the gentamicin-induced renal damage. It was noticed that gentamicin-induced lesions subsided with RB treatment in dose dependent manner confirming the nephroprotective effect of RB.

Hydro-alcoholic extract (70% methanol extract) of stems of *Caralluma umbellata* Haw significantly reduced the renal damage caused by cisplatin and gentamicin at a dose of 500 mg/kg^33^.

## Material and method

### Plant materials

Aerial parts of Caralluma quadrangula (Forssk.) N.E.Br. (syn. Monolluma quadrangula (Forssk.) Plowes) were collected from Al-Taif Governorate, Saudi Arabia (2022), and all necessary consent was obtained from the Al-Taif Governorate before collecting plant samples. The plant authentication was done by a staff member at the Taxonomy Department at the Faculty of Science at King Abdulaziz University and a specimen (CQ 1027-B) was deposited in the herbarium of College of Pharmacy, King Abdulaziz University, Jeddah, Saudi Arabia.

### Isolation of russelioside B

**Fig. 11:**
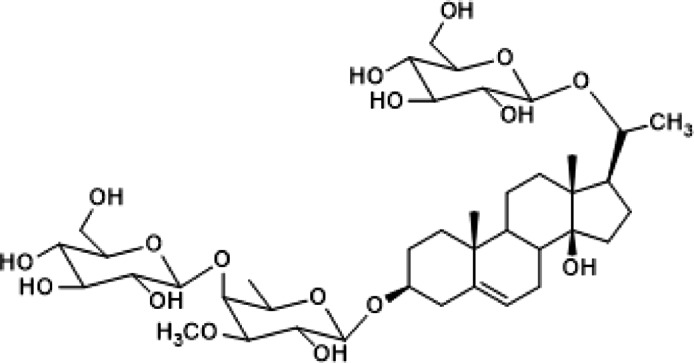
Chemical structure of Russelioside B.

The air-dried powdered *C. quadrangula* aerial parts (480 g) were extracted with methanol and the methanolic extract was evaporated till dryness to yield a brown residue (82 g). The methanolic extract (65 g) was fractionated into chloroform fraction (8.8 g), and *n*-butanol fraction (35.8 g). The *n*-butanol fraction was chromatographed on a silica gel column according to the procedures described previously to isolate russelioside B (calogenin 20-*O*-*β*-D-glucopyranosyl-3-*O*-[*β*-D-glucopyranosyl-(1 → 4)-β-D-(3-*O*-methyl-6-deoxy) galactoside])^8^. The structure of RB (Fig. 11) was confirmed by superimposed IR and by comparison of its ^1^H-NMR and ^13^C-NMR (Suppl. Fig. S1 and S2) with those reported in the literature ^14^.

### Animals model

Twenty male Sprague–Dawley rats were used in this experiment, weighing 180–200 g. They were obtained seven days before proceeding with the experiment from the Egyptian company for the production of vaccines, sera, and drugs (VACSERA). They were housed in rat cages with appropriate conditions. The rats had free access to food and water throughout the entire experiment.

### Experimental design

All experiments were conducted in accordance with relevant guidelines and regulations. Experiments on rats were carried out in accordance with ARRIVE 2.0 guidelines and were approved by the Institutional Animal Care and Use Committee (IACUC) in the Faculty of Veterinary Medicine, Cairo University under the code (Vet CU 0,909,202,775).

Animals were allocated randomly into 4 groups (5 animals per group); G1 was the **(Normal group)** that didn’t receive any medication. G2 was the (**Gentamicin)** group that received gentamicin only (Garamycin®- Memphis Pharmacology company (100 mg/kg, i.p.) for 7 consecutive days^2,34^. G3 **(Genta + 50RB)** was the group given a low dose of RB (50 mg/kg B.wt, orally) for 3 days, followed by the same dose along with gentamicin injection for 7 consecutive days. G4 was the **(Genta + 75RB)** group, that was given a high dose of RB (75 mg/kg B wt, orally) for 3 days, followed by the same dose along with gentamicin injection for 7 consecutive days.

A summarization of the experimental design and the assessments used for the evaluation of the nephroprotection (Fig. 10).

### Sample collection

At the end of the 10th day, each animal was weighed individually. Blood was collected by puncturing the inner canthus of the eye (under anesthesia using Isoflurane by inhalation ), and serum was separated. Afterward, animals were euthanized by cervical dislocation^35^, and kidneys were removed at once for gross examination and weighting. One of the kidneys was frozen for biochemical testing and antioxidants evaluation, while the other one was preserved in 10% neutral buffered formalin for histopathological examination.

### Gross examination and kidney weight-to-body weight ratio calculation

Photographs of the kidneys were taken from each experimental group. Additionally, the kidney weight to the body weight ratio (KW/BW) was calculated as kidney weight divided by 100 g of body weight (KW/100 g BW)^17^.

### Evaluation of kidneys’ function markers

The separated serum was used to measure creatinine and blood urea nitrogen (BUN). The assessment was performed using kits obtained from Biodiagnostic, Dokki, Giza, Egypt, following the manufacturer’s instructions.

### Evaluation of oxidative stress index

The frozen kidney tissue samples were washed with EDTA. Then, the tissue was homogenized in 1.15% KCl buffer and centrifuged at 4000 rpm for 10 min. The resulting supernatant was collected for the assessment of superoxide dismutase (SOD), malondialdehyde (MDA), and nitric oxide (NO) using kits from Biodiagnostic, Dokki, Giza, Egypt, following the manufacturer’s instructions.

### Quantitative real-time PCR (qRT-PCR) for IGF, iNOS, TNF-α and 2

Total RNA was extracted from kidney tissue using QIAmp miRNAsy mini kit (QIAGEN, Hilden, Germany) according to the manufacturer instructions. The concentration and purity of the total RNA samples were obtained using a nanodrop ND-1000 spectrophotometer. The isolated RNA was used for cDNA synthesis using M-MLV reverse transcriptase (Fermentas, EU). Real-time PCR (qPCR) was carried out using the reaction mixture of cDNAs, iQ SYBR Green Premix (Bio–Rad 170–880, USA), and 0.5 mM of each primer (Insulin-like growth factor 1 (IGF-1), inducible Nitric oxide synthase (iNOS), B-cell lymphoma 2 (Bcl2) and B-actin as an internal control). PCR amplification and analysis were achieved using Bio– Rad iCycler thermal cycler and the MyiQ real time PCR detection system. The primers and their sequence published in GenBank are shown in Table 1. Each assay includes triplicate samples of each tested cDNA and no-template negative control and the ΔCT value is calculated according to^36^ through the subtraction of the B- actin CT from each gene’s CT; in which CT is the cycle number where detectable signals are obtained.

**Table 1:** Primer Sequences of IGF-1, iNOS, Bcl2 and B- actin Genes.

Gene	Primer	Sequence	Accession number	Amplicon (bp)	Reference
IGF-1	Forward	TCTCCTAGTCCCTGCCTCTT	XM_039078402.1	183	^38^
	Reverse	TCTGTGAAGGAAGCGGCTTA			
iNOS	Forward	GTTTGACCAGAGGACCCAGA	XM_006246949.3	175	^39^
	Reverse	GTGAGCTGGTAGGTTCCTGT			
	Reverse	GGAACAGTCTGGGAAGCTCT			
Bcl2	Forward	GATTTCTCCTGGCTGTCTCTGAA	NM_016993.2	254	^40^
	Reverse	GTGTGTGTGTGTGTGTGTGTG			
B- Actin (reference gene)	Forward	AGGCTGTGTTGTCCCTGTATG	NM_031144.3	275	^41^
	Reverse	GGCCATCTCTTGCTCGAAGT			

### Histopathological examination

The kidney tissues were fixed in 10% neutral buffered formalin. They were sectioned into 5-μm-thick sections, after dehydration, clearance and paraffin wax embedding. Tissue sections were rehydrated and routinely stained with hematoxylin and eosin (H&E)^37^. Tissue slides were examined with Leica DM4 B light digital microscope (Germany) and photomicrographs were taken with a Leica DMC 4500 digital camera (Germany).

Histological tissue scoring was performed according to the method described by^32^. All assessed features were graded on a scale from 0 to 4, as follows: 0 = no damage, 1 = mild damage, 2 = moderate damage, 3 = damage less than 25%, and 4 = damage between 25 and 50%. A score of 4 was also given for damage exceeding 50%.The renal cortex was assessed for degeneration and necrosis, cast formation, inflammation, and congestion. Ten random microscopic fields were assessed from each individual kidney sample i.e.: (50 number in each group).

### Immunohistochemistry

The kidney’s tissue was sectioned into 5-μm thickness for immune histochemistry and loaded on positively charged glass slides. It was then rehydrated and put in a microwave oven to retrieve the epitopes. Next, primary antibodies were added as detailed in Table 2 and the tissue sections were incubated for an hour at room temperature in a humid chamber. After washing, hydrogen peroxidase was added for blocking endogenous peroxidases and then the slides were washed. HRP-labelled secondary detection kit (BioSB, USA) was used as manufacturer instructions to develop the reaction. The primary antibody step was deleted for negative control slides. The specific brown color of positive expression was quantified as area percentage in five random microscopic fields in each group.

**Table 2:** Primary antibodies used for immunohistochemistry.

Primary antibodies	Abbreviation	Cat. Number and manufacturing company	Dilution
Anti-silent mating type information regulation 1	Anti-SIRT1	13,161–1-AP Proteintech, Germany	1:300
Anti-NAD(P)H: quinone acceptor oxidoreductase 1	Anti-NQO1	11,451–1-AP Proteintech, Germany	1:300
Anti-nuclear factor erythroid 2–related factor 2	Anti-Nrf2	16,396–1-AP Proteintech, Germany	1:300
Anti-tumor necrosis factor alpha	Anti-TNF-α	sc-52746 SantaCruz, Biotechnology, Inc	1:200
Anti-nuclear factor kappa-light-chain-enhancer of activated B cells	Anti-NF-κB	sc-8008 SantaCruz, Biotechnology, Inc	1:200
Anti-Bcl-2–associated X protein	Anti-BAX	sc-7480 SantaCruz, Biotechnology, Inc	1:100

### Statistical analysis

All data were presented statically as mean ± standard error (SE). One-way ANOVA test was applied and Kruskal–Wallis test was used for histological scoring. Differences between groups were considered significant when *p* < 0.05. GraphPad Prism 8 was used.

## Conclusion

Our findings confirmed the efficacy of Russelioside B, isolated from C*ralluma quadrangula* as a nephroprotective agent against gentamicin-induced acute renal injury. RB improved the oxidative status of renal tissue, suppressed inflammation and protected the renal tissue against apoptosis. RB also improved gentamicin-induced histopathological alterations.

## Supplementary Information

Below is the link to the electronic supplementary material.

Supplementary Material 1

## Data Availability

All data used during the current study is available in the article.
