# Supplementary figures and images for: The nephroprotective potential of russelioside B isolated from Caralluma quadrangula in gentamicin-induced acute kidney injury via modulation of SIRT-1 pathway
Source: Sci Rep. 2025 Dec 13;15:43829. doi: 10.1038/s41598-025-29874-7 (PMC12705675; doi:10.1038/s41598-025-29874-7)

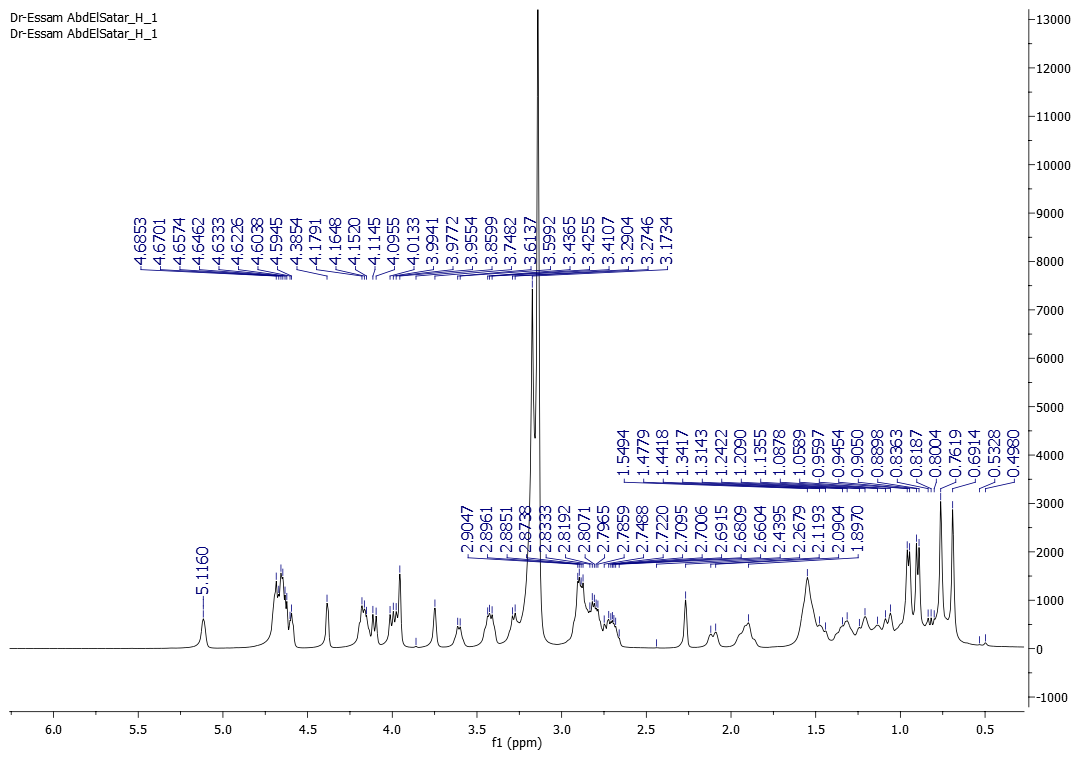


**Fig. S1.** 1H-NMR spectrum of of russelioside B


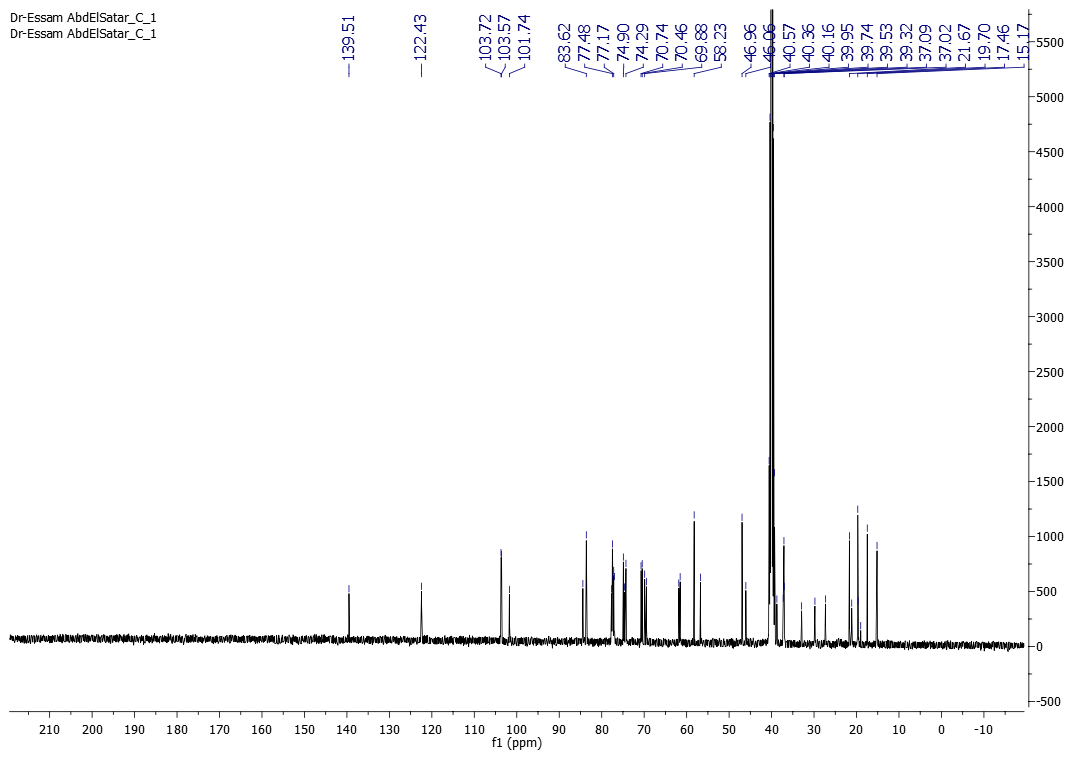


**Fig. S2.** 13C-NMR spectrum of of russelioside B

Supplement: Supplementary file 1 — Supplementary Material 1 [file 41598_2025_29874_MOESM1_ESM.docx]
